# Urticaria on Arms and Chest

**Published:** 1904-12

**Authors:** 


### URTICARIA ON ARMS AND CHEST.

The following treatment rarely fails to give relief:

Sponge the parts affected with a 2 per cent. solution of Hux-Sal (Antiseptic Salt) or apply as compresses to allay itching. Allow the body to dry and then apply with some friction.

℞ Mentholi .......................................... ʒ i.

Spt. Vini Rect.......................................... ℥ i.

Betul-ol (Oleo-Methyl-Salicy. Comp.)............... ʒ iv.

M. ft. applic.

(For external use only.)

